# Retraction: Independent and combined associations of sleep duration and sleep quality with common physical and mental disorders: Results from a multi-ethnic population-based study

**DOI:** 10.1371/journal.pone.0359897

**Published:** 2026-10-06

**Authors:** The PLOS One Editors

After publication of this article [1], the corresponding author notified *PLOS One* that errors occurred in the coding of the sociodemographic and poor sleep variables, which affects the statistical results in all tables in [1], as well as the text describing the results throughout the article.

The *PLOS One* Editors concluded that these errors affect some of the conclusions of the article. Specifically, after the coding error is corrected, the revised analyses show sleep duration is not associated with any condition. After consultation with a member of the Editorial Board, the *PLOS One* Editors concluded that the remainder of the conclusions in [1] are mainly unaffected by the error.

The *PLOS One* Editors consider that a key conclusion of this article is no longer upheld, and the coding error substantively affects the reported statistical results in [1]. To address this concern, the *PLOS One* Editors retract this article.

All authors either did not respond directly to the final editorial decision or could not be reached.
